# Supplementary material for: Efficacy and Safety of Trans-Arterial Yttrium-90 Radioembolization in Patients with Unresectable Liver-Dominant Metastatic or Primary Hepatic Soft Tissue Sarcomas
Source: Cancers (Basel). 2022 Jan 10;14(2):324. doi: 10.3390/cancers14020324 (PMC8774147; doi:10.3390/cancers14020324)
Supplement: Supplementary file 1 [file cancers-14-00324-s001.zip › cancers-1538800-supplementary.pdf]

Table S1. Effect of other cancer-directed therapies on radiologic tumor response post-TARE measured by RECIST criteria.

	Chemotherapy Post-TARE Yes	Chemotherapy Post-TARE No	*p*-value (χ^2)
**3 months post-TARE (RECIST)**			
DC (CR+PR+SD)	71.4% (15/21)	77.8% (7/9)	.71
ORR (CR+PR)	13.3% (3/21)	56.7% (1/9)	.81
PD	28.6% (6/21)	22.2% (2/9)	
**6 months post-TARE (RECIST)**			
DC (CR+PR+SD)	58.8% (10/17)	75.0% (6/8)	.43
ORR (CR+PR)	23.5% (4/17)	12.5% (1/8)	.52
PD	41.2% (7/17)	25.0% (2/8)	
**9 months post-TARE (RECIST)**			
DC (CR+PR+SD)	30.8% (4/13)	100.0% (2/2)	.06
ORR (CR+PR)	7.7% (1/13)	50.0% (1/2)	.10
PD	69.2% (9/13)	0.0% (0/2)	
	**Chemotherapy Pre-TARE Yes**	**Chemotherapy Pre-TARE No**	***p*-value (χ^2)**
**3 months post-TARE (RECIST)**			
DC (CR+PR+SD)	75.0% (18/24)	66.7% (4/6)	.67
ORR (CR+PR)	16.7% (4/24)	0.0% (0/6)	.28
PD	25.0% (6/24)	33.3% (2/6)	
**6 months post-TARE (RECIST)**			
DC (CR+PR+SD)	63.2% (12/19)	66.7% (4/6)	.87
ORR (CR+PR)	21.0% (4/19)	16.7% (1/6)	.81
PD	36.8% (7/19)	33.3% (2/6)	
**9 months post-TARE (RECIST)**			
DC (CR+PR+SD)	45.4% (5/11)	25.0% (1/4)	.47
ORR (CR+PR)	18.2% (2/11)	0.0% (0/4)	.35
PD	54.6% (6/11)	75.0% (3/4)	
	**Liver-directed therapy Pre-TARE Yes**	**Liver-directed therapy Pre-TARE No**	***p*-value (χ^2)**
**3 months post-TARE (RECIST)**			
DC (CR+PR+SD)	50.0% (1/2)	75.0% (21/28)	.43
ORR (CR+PR)	0.0% (0/2)	14.3% (4/28)	.56
PD	50.0% (1/2)	25.0% (7/28)	
**6 months post-TARE (RECIST)**			
DC (CR+PR+SD)	50.0% (1/2)	65.2% (15/23)	.66
ORR (CR+PR)	0.0% (0/2)	21.7% (5/23)	.46
PD	50.0% (1/2)	34.8% (8/23)	
**9 months post-TARE (RECIST)**			
DC (CR+PR+SD)	0.0% (0/3)	50.0% (6/12)	.11
ORR (CR+PR)	0.0% (0/3)	16.7% (2/12)	.44
PD	100.0% (3/3)	50.0% (6/12)	
	**Liver Surgery Pre-TARE Yes**	**Liver Surgery Pre-TARE No**	***p*-value (χ^2)**
**3 months post-TARE (RECIST)**			

DC (CR+PR+SD)	50.0% (4/8)	81.8% (18/22)	.08
ORR (CR+PR)	0.0% (0/8)	18.2% (4/22)	.19
PD	50.0% (4/8)	18.2% (4/22)	
**6 months post-TARE (RECIST)**			
DC (CR+PR+SD)	55.6% (5/9)	68.8% (11/16)	.50
ORR (CR+PR)	11.1% (1/9)	25.0% (4/16)	.40
PD	44.4% (4/9)	31.2% (5/16)	
**9 months post-TARE (RECIST)**			
DC (CR+PR+SD)	40.0% (2/5)	40.0% (4/10)	>0.9
ORR (CR+PR)	0.0% (0/5)	20.0% (2/10)	.28
PD	60.0% (3/5)	60.0% (6/10)	

	**Radiotherapy Pre-TARE Yes**	**Radiotherapy Pre-TARE No**	***p*-value (χ^2)**
**3 months post-TARE (RECIST)**			
DC (CR+PR+SD)	78.6% (11/14)	68.8% (11/16)	.54
ORR (CR+PR)	21.4% (3/14)	6.2% (1/16)	.22
PD	21.4% (3/14)	31.2% (5/16)	
**6 months post-TARE (RECIST)**			
DC (CR+PR+SD)	70.0% (7/10)	60.0% (9/15)	.60
ORR (CR+PR)	20.0% (2/10)	20.0% (3/15)	>0.9
PD	30.0% (3/10)	40.0% (6/15)	
**9 months post-TARE (RECIST)**			
DC (CR+PR+SD)	33.3% (2/6)	44.4% (4/9)	.66
ORR (CR+PR)	16.7% (1/6)	11.1% (1/9)	.75
PD	66.7% (4/6)	55.6% (5/9)	

TARE, trans-arterial radioembolization; RECIST, Response Evaluation Criteria in Solid Tumors; DC, disease control; CR, complete response; PR, partial response; SD, stable disease; PD, disease progression; ORR, objective response rate; (χ2), Chi-Square Test.

Table S2. Effect of other cancer-directed therapies on radiologic tumor response post-TARE measured by mRECIST criteria.

	Chemotherapy Post-TARE<br>Yes	Chemotherapy Post-TARE<br>No	*p*-value (χ^2)
**3 months post-TARE (mRECIST)**			
DC (CR+PR+SD)	80.9% (17/21)	77.8% (7/9)	.84
ORR (CR+PR)	57.1% (12/21)	55.6% (5/9)	.94
PD	19.1% (4/21)	22.2% (2/9)	
**6 months post-TARE (mRECIST)**			
DC (CR+PR+SD)	64.7% (11/17)	75.0% (6/8)	.60
ORR (CR+PR)	52.9% (9/17)	50.0% (4/8)	.89
PD	35.3% (6/17)	25.0% (2/8)	
**9 months post-TARE (mRECIST)**			
DC (CR+PR+SD)	38.5% (5/13)	100.0% (2/2)	.10
ORR (CR+PR)	30.8% (4/13)	100.0% (2/2)	.06
PD	61.5% (8/13)	0.0% (0/2)	
	**Chemotherapy Pre-TARE<br>Yes**	**Chemotherapy Pre-TARE<br>No**	***p*-value (χ^2)**
**3 months post-TARE (mRECIST)**			
DC (CR+PR+SD)	83.3% (20/24)	66.7% (4/6)	.36
ORR (CR+PR)	54.2% (13/24)	66.7% (4/6)	.58
PD	16.7% (4/24)	33.3% (2/6)	
**6 months post-TARE (mRECIST)**			
DC (CR+PR+SD)	68.4% (13/19)	66.7% (4/6)	.93
ORR (CR+PR)	47.4% (9/19)	66.7% (4/6)	.40
PD	31.6% (6/19)	33.3% (2/6)	
**9 months post-TARE (mRECIST)**			
DC (CR+PR+SD)	55.6% (6/11)	25.0% (1/4)	.31
ORR (CR+PR)	45.4% (5/11)	25.0% (1/4)	.47
PD	45.4% (5/11)	75.0% (3/4)	
	**Liver-directed therapy Pre-TARE<br>Yes**	**Liver-directed therapy Pre-TARE<br>No**	***p*-value (χ^2)**
**3 months post-TARE (mRECIST)**			
DC (CR+PR+SD)	50.0% (1/2)	82.1% (23/28)	.27
ORR (CR+PR)	50.0% (1/2)	57.1% (16/28)	.84
PD	50.0% (1/2)	17.9% (5/28)	
**6 months post-TARE (mRECIST)**			
DC (CR+PR+SD)	50.0% (1/2)	69.6% (16/23)	.56
ORR (CR+PR)	50.0% (1/2)	52.2% (12/23)	.95
PD	50.0% (1/2)	30.4% (7/23)	
**9 months post-TARE (mRECIST)**			
DC (CR+PR+SD)	0.0% (0/3)	58.3% (7/12)	.07
ORR (CR+PR)	0.0% (0/3)	50.0% (6/12)	.11
PD	100.0% (3/3)	41.7% (5/12)	
	**Liver Surgery Pre-TARE<br>Yes**	**Liver Surgery Pre-TARE<br>No**	***p*-value (χ^2)**
**3 months post-TARE (mRECIST)**			
DC (CR+PR+SD)	50.0% (4/8)	90.9% (20/22)	.01

ORR (CR+PR)	50.0% (4/8)	59.1% (13/22)	.65
PD	50.0% (4/8)	9.1% (2/22)	
**6 months post-TARE (mRECIST)**			
DC (CR+PR+SD)	55.6% (5/9)	75.0% (12/16)	.31
ORR (CR+PR)	44.4% (4/9)	56.2% (9/16)	.57
PD	44.4% (4/9)	25.0% (4/16)	
**9 months post-TARE (mRECIST)**			
DC (CR+PR+SD)	40.0% (2/5)	50.0% (5/10)	.71
ORR (CR+PR)	40.0% (2/5)	40.0% (4/10)	>0.9
PD	60.0% (3/5)	50.0% (5/10)	
	**Radiotherapy Pre-TARE Yes**	**Radiotherapy Pre-TARE No**	***p*-value (χ^2)**
**3 months post-TARE (mRECIST)**			
DC (CR+PR+SD)	85.7% (12/14)	75.0% (12/16)	.46
ORR (CR+PR)	64.3% (9/14)	50.0% (8/16)	.43
PD	14.3% (2/14)	25.0% (4/16)	
**6 months post-TARE (mRECIST)**			
DC (CR+PR+SD)	70.0% (7/10)	66.7% (10/15)	.86
ORR (CR+PR)	60.0% (6/10)	46.7% (7/15)	.51
PD	30.0% (3/10)	33.3% (5/15)	
**9 months post-TARE (mRECIST)**			
DC (CR+PR+SD)	33.3% (2/6)	55.6% (5/9)	.39
ORR (CR+PR)	33.3% (2/6)	44.4% (4/9)	.66
PD	66.7% (4/6)	44.4% (4/9)	

TARE, trans-arterial radioembolization; mRECIST, Modified Response Evaluation Criteria in Solid Tumors; DC, disease control; CR, complete response; PR, partial response; SD, stable disease; PD, disease progression; ORR, objective response rate; (χ2), Chi-Square Test.

Table S3. Univariate Analysis of Overall Survival after TARE based on mRECIST response at 3-, 6- and 9-months post-procedure.

	Number (%)	Kaplan Meier<br>Median OS (months, 95%CI)	*p*-Value<br>(Log-rank)	Cox Proportional Hazards<br>Hazard Ration (95%CI)	*p*-Value<br>(Cox)
**mRECIST response 3-months**					
DC	24 (68.6%)	22(10.9–33.0)	.089	0.44(0.17–1.2)	.104
PD	6 (17.1%)	10(0.0–22.0)			
**mRECIST response 6-months**					
DC	17 (48.6%)	40(17.8–62.2)	**.003**	0.23(0.08–0.68)	**.008**
PD	8 (22.8%)	16(6.3–25.7)			
**mRECIST response 9-months**					
DC	8 (22.8%)	94(5.2–182.8)	.125	0.29(0.05–1.5)	.146
PD	7 (20.0%)	33(14.9–51.0)			

TARE, trans-arterial radioembolization; mRECIST, Modified Response Evaluation Criteria in Solid Tumors; DC, disease control; PD, disease progression; 95%CI, 95% Confidence Interval; OS, overall survival.
